# Changes in IP3 Receptor Expression and Function in Aortic Smooth Muscle of Atherosclerotic Mice

**DOI:** 10.1159/000461581

**Published:** 2017-04-01

**Authors:** Marie-Ann Ewart, Azizah Ugusman, Anisha Vishwanath, Tarek A.M. Almabrouk, Husam Alganga, Omar J. Katwan, Pavlina Hubanova, Susan Currie, Simon Kennedy

**Affiliations:** ^a^Institute of Cardiovascular and Medical Sciences, College of Medical, Veterinary and Life Sciences, University of Glasgow, Glasgow, UK; ^b^Strathclyde Institute of Pharmacy and Biomedical Sciences, Strathclyde University, Glasgow, UK; ^c^Department of Physiology, Faculty of Medicine, National University of Malaysia Medical Centre, Kuala Lumpur, Malaysia

**Keywords:** Peroxynitrite, IP3 receptor, Potassium channel, Atherosclerosis, Aorta

## Abstract

Peroxynitrite is an endothelium-independent vasodilator that induces relaxation via membrane hyperpolarization. The activation of IP3 receptors triggers the opening of potassium channels and hyperpolarization. Previously we found that relaxation to peroxynitrite was maintained during the development of atherosclerosis due to changes in the expression of calcium-regulatory proteins. In this study we investigated: (1) the mechanism of peroxynitrite-induced relaxation in the mouse aorta, (2) the effect of atherosclerosis on relaxation to peroxynitrite and other vasodilators, and (3) the effect of atherosclerosis on the expression and function of the IP3 receptor. Aortic function was studied using wire myography, and atherosclerosis was induced by fat-feeding ApoE^−/−^ mice. The expression of IP3 receptors was studied using Western blotting and immunohistochemistry. Relaxation to peroxynitrite was attenuated by the IP3 antagonists 2-APB and xestospongin C and also the K_v_ channel blocker 4-aminopyridine (4-AP). Atherosclerosis attenuated vasodilation to cromakalim and the AMPK activator A769662 but not peroxynitrite. Relaxation was attenuated to a greater extent by 2-APB in atherosclerotic aortae despite the reduced expression of IP3 receptors. 4-AP was less effective in ApoE^−/−^ mice fat-fed for 4 months. Peroxynitrite relaxation involves an IP3-induced calcium release and K_V_ channel activation. This mechanism becomes less important as atherosclerosis develops, and relaxation to peroxynitrite may be maintained by increased calcium extrusion.

## Introduction

The cytosolic concentration of calcium is the primary determinant of the contractile state of the vascular smooth muscle cell (VSMC). Cytosolic calcium levels can be increased via entry across the plasma membrane through voltage-operated calcium channels [1] or non-selective cation channels or via store-operated calcium entry (SOCE) [2]. An increase in calcium can also occur via release from the intracellular sarcoplasmic reticulum (SR) through intracellular calcium channels, namely IP3 receptors (IP3R) and ryanodine receptors.

Direct activation of IP3R present on the SR by the second messenger inositol 1,4,5-triphosphate results in calcium release and increased global cytosolic Ca^2+^. Indirect activation of ryanodine receptors resulting from a global increase in cytosolic Ca^2+^ results in a further Ca^2+^ release (albeit a small release in comparison to IP_3_R contribution) and the combined effect leads to smooth muscle cell (SMC) contraction. Interestingly, in the last 10 years, it has become apparent that IP3R can communicate locally with plasma membrane channels such as transient receptor potential canonical 3 and large-conductance Ca^2+^-activated potassium channels (BK_Ca_) [reviewed in 3].

Activation of IP3R renders the BK_Ca_ channel more sensitive to calcium in vascular smooth muscle [4], leading to membrane hyperpolarization which may serve to limit contractility in response to IP3R activation. BK_Ca_ channels are abundantly expressed in blood vessels and it has been suggested that this may be the switch that decides whether vasoactive factors induce vasoconstriction (via Ca^2+^ signalling) or vasorelaxation (via membrane hyperpolarization). Indeed, a recent study found that IP3 activated BK_Ca_ in porcine coronary SMC with a resultant decrease in vessel tone [5]. Taken together, these studies highlight the pivotal role that IP3R play in modulating Ca^2+^ handling and vascular contractility in the healthy animal.

Peroxynitrite (ONOO^−^) is a reaction product of nitric oxide and superoxide and as such it has been detected in increased quantities in the arterial wall of atherosclerotic mice [6] and also in the plasma of human subjects with acute coronary syndrome [7]. In healthy vessels, ONOO− induces endothelium-independent relaxation [8] through elevation of cGMP levels, membrane hyperpolarization, and direct activation of myosin phosphatase activity in smooth muscle. Vasodilation also involves calcium regulatory proteins, with increases in SERCA activity seen in artery homogenates in response to ONOO^−^[9]. However, to date there is no information on how IP3R-induced calcium release and membrane hyperpolarization via potassium channel activation are involved in ONOO^−^-induced relaxation.

In a previous study we demonstrated that arterial relaxation to ONOO^−^ was maintained in atherosclerotic ApoE^−/−^ mice fed a high-fat diet for up to 4 months, despite downregulation of SERCA expression and function in VSMC. We concluded that upregulation at the protein level of the calcium-extruding PMCA compensates for the effect of atherosclerosis on SERCA and maintains vascular relaxation under atherogenic conditions. However, the role of IP3R in maintaining relaxation to ONOO^−^ in this pathology is not well understood. In hypercholesterolaemic mice without evidence of atherosclerotic plaques, the intracellular calcium [Ca^2+^]_i_ in aortic VSMC was increased due to augmented IP3R-mediated SOCE rather than an enhanced SR calcium release [10], and this may be related to changes in plasma membrane cholesterol in response to raised plasma lipid levels [11]. Although very few other studies have examined IP3R function in atherosclerosis, there is evidence in hypertensive mice and rats that IP3R transcript and protein levels are raised in mesenteric resistance vessels [12]. In hypertensive animals, pharmacological experiments have also revealed that IP3-mediated calcium release contributes more to contraction compared to control animals. Increased pulsatile pressure applied to rat VSMC in vitro also increased cell migration and this occurred via IP3R since it was blocked by the IP3R blockers 2-APB and xestospongin C [13]. These studies support the likelihood that IP3R function in vascular smooth muscle may be altered under pathophysiological conditions and may result in altered SOCE as well as SR Ca^2+^ handling. Changes in SOCE in pathological conditions may also affect other Ca^2+^ channels. For example, recently discovered channels such as the Orai family, which regulates calcium entry in response to store depletion, may be particularly important in disease states [14].

In this study, we investigated the mechanism of ONOO^−^ relaxation in mouse aortic rings and also studied the effect of developing atherosclerosis on IP3R expression and vascular function in response to ONOO^−^ and other endothelium-independent vasodilators.

## Materials and Methods

### Animal Model and Artery Preparation

The mice used in this study were housed at the University of Glasgow and maintained on 12-h cycles of light and dark and at ambient temperature. Initial experiments to study the mechanism of relaxation induced by ONOO^−^ used male C57BL/6 mice (18–23 g, supplied by Harlan). To study the effect of developing atherosclerosis, male ApoE^−/−^ mice (17–38 g, bred in-house) and genetic background control mice (C57BL/6; 18–34 g) were used. C57BL/6 mice were fed a standard chow diet while ApoE^−/−^ mice commenced a high-fat diet (21% lard and 0.15% cholesterol; SDS) at 8 weeks of age which was continued for between 2 and 4 months. Age-matched C57 mice were used in all experiments. To assess the effects of ApoE gene knockout alone, some experiments also used chow-fed ApoE^−/−^ mice. Procedures conformed to the Guide for the Care and Use of Laboratory Animals published by the US National Institutes of Health (NIH publication No. 85-23, revised in 1996) and Directive 2010/63/EU of the European Parliament. Mice were terminally anaesthetized via intraperitoneal injection of sodium pentobarbital (200 mg/mL) and the thoracic aorta was removed to ice cold oxygenated (95% O_2_:5% CO_2_) Krebs' solution for preparation.

### Small Vessel Wire Myography

The thoracic aorta was cleaned of all fat and connective tissue and cut into 2-mm rings. In some experiments the endothelium was left intact but in the majority of cases it was removed by gently rubbing the lumen of the vessel with a piece of fine wire. Artery rings were mounted on 2 stainless steel pins in a 4-channel wire myograph (Danish Myo Technology), set to an optimum tension of 9.8 mN [15], and allowed to equilibrate for at least 30 min before use. Vessels were bathed in Krebs' buffer with the following composition: 118 mM NaCl, 4.7 mM KCl, 1.2 mM MgSO_4_, 25 mMNaHCO_3_, 1.03 mM KH_2_PO_4_, 11 mM glucose, and 2.5 mM CaCl_2_ at 37°C and gassed continuously with 95% O_2_ and 5% CO_2_. Reproducible responses to 40 mM KCl (typically 2 separate additions per ring) were obtained and then rings were contracted to 30 nM 9,11-dideoxy-9α,11α-methanoepoxy prostaglandin F_2α_ (U46619; Tocris) and successful removal of the endothelium was confirmed by the lack of (<10%) a vasodilator response to 10^−6^M acetylcholine. Rings were then washed, antagonists were added, and contraction was induced a second time by addition of 30 nM U46619, and once the level of contractile force had stabilized (typically 15–20 min) the experiments were commenced. This allowed the contractile responses and the effect of 2-APB in C57BL/6 and ApoE^−/−^ mice to be compared.

In pre-contracted rings, dose response curves were constructed to 3 different vasodilators: (1) ONOO^−^ (Calbiochem) used in the range 1 × 10^−6^−5 × 10^−4^M, and ONOO^−^ was diluted in argon-purged dH_2_O and kept in the dark at 4°C; (2) cromakalim (Sigma-Aldrich), an activator of K_ATP_ channels on VSMC, was used in the concentration range 1 × 10^−9^-5 × 10^−6^M, and cromakalim was dissolved in DMSO and diluted in Krebs'; and (3) the AMPK activator A769662 [6,7-dihydro-4-hydroxy-3-(2'-hydroxy[1, 1'-biphenyl]-4-yl)-6-oxo-thieno(2, 3-*b*)pyridine-5-carbonitrile; Tocris]. A769662 was dissolved in DMSO, diluted in Krebs', and used in the range 1 × 10^−6^-5 × 10^−4^M[16]. All 3 vasodilators were added at 10-min intervals. To study the involvement of the IP3R, rings were pre-incubated with the IP3R antagonists 2-aminoethoxydiphenyl borate (2-APB; 60 µM; Tocris) or xestospongin C (0.5 or 5 µM; Cayman Chemical Company) for 30 min before the addition of vasodilators. Xestospongin C is a much more potent and selective IP3R antagonist that is effective at low micromolar concentrations [17].

To study the involvement of potassium channels which may be activated by a IP3R-induced calcium release, aortic rings were incubated with: the general Ca^2+^-activated potassium channel blocker tetraethylammonium chloride (TEA, 1 mM; Sigma-Aldrich), the K_v_ channel blocker 4-aminopyridine (4-AP, 1 mM; Sigma-Aldrich), the ATP-sensitive potassium channel blocker glibenclamide (0.3 µM; Sigma-Aldrich), or the BK_Ca_-selective channel blocker iberiotoxin (100 nM; Latoxan, Portes les Valance, France). All were incubated for 30 min before contraction to U46619. For all experiments, data were expressed as a percentage relaxation of the U46619-induced tone.

### Histological Analysis

Cleaned thoracic aortae were fixed in neutral-buffered formalin and embedded in paraffin and 4-µm sections were cut on a rotary microtome. IP3R (IP3R1) was detected in sections using a rabbit polyclonal IP3R (type 1) antibody (1:1,000 dilution, Ab5804; Abcam) and visualized using biotin-labelled secondary antibody-streptavidin-HRP complexes and a DAB (3,3′ diaminobenzidine) chromogenic substrate (Vector Laboratories). Images were analysed blindly by 2 independent observers and the degree of staining was quantified using ImageJ software.

### Protein Expression/Immunoblotting

Denuded aortae were pulverized in liquid nitrogen, re-suspended in ice-cold cell lysis buffer (50 mM Tris [pH 7.4], 50 mM NaF, 1 mM Na_4_PPi, 1 mM EGTA, 1 mM EDTA, 1% Triton X-100, 1 mM DTT, and 1% cocktail of protease inhibitors) and protein concentrations determined using Coomassie Plus Protein Assay Reagent (Perbio, USA). Homogenates were run at several protein concentrations (2.5, 5 and 7.5 µg/lane) on NuPAGE Novex 4–12% Bis-Tris mini gels (Life Technologies) and transferred to a nitrocellulose membrane, and IP3R was detected using rabbit polyclonal anti-IP3R1 (No. 3763 used at 1:1,000; Cell Signaling Technology). Large conductance calcium-activated potassium channels (BK_Ca_) in lysates were detected using an anti BK_Ca_ mouse monoclonal antibody (1:500 dilution, ab99046; ABcam). Secondary HRP-conjugated antibodies were either goat anti-rabbit HRP conjugated for IP3 detection (ab6721; Abcam) or rabbit anti-mouse HRP conjugated for BK_Ca_ detection (ab6728; Abcam). IP3R and BK_Ca_ protein abundance was quantified using Quantity One software (BioRad). Expression was normalized to GAPDH (1:40,000. No. G8795; Sigma-Aldrich) or α-actin (1:1,000, No. 14958; Cell Signaling Technology).

### Statistical Analysis

All results are presented as means ± SEM, and *n* represents the number of mice used for each experiment. Myography data were analysed via GraphPad Prism software and significance was determined using 2-way ANOVA which compares the full dose-response curves. The area under the curve was used to analyse differences in the inhibitory effect of 2-APB in control and ApoE^−/−^ mice [18]. Student's *t* tests (unpaired) were performed on IP3R or BK_Ca_ expression data. In all cases, *p* < 0.05 was considered statistically significant.

## Results

### Relaxation to ONOO^*−*^ in C57BL/6 Aortic Rings

**Fig. 1:**
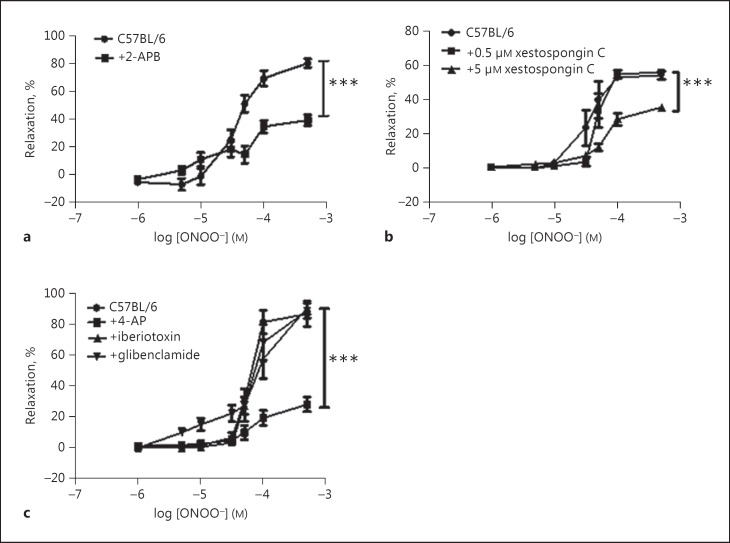
**a** In denuded C57BL/6 mouse aortic rings, the IP3 receptor antagonist 2-APB (60 µM) significantly reduced relaxation to peroxynitrite. **b** Another IP3 receptor antagonist, xestospongin C showed a dose-dependent effect, blocking relaxation to peroxynitrite at 5 µM. **c** Relaxation to peroxynitrite was significantly reduced by 4-AP but not other potassium channel blockers. *** *p* < 0.001 vs C57BL/6 control group, *n* > 5 for all groups.

Peroxynitrite induced a dose-dependent vasodilation of a similar magnitude in endothelium-intact and denuded thoracic aortae from C57BL/6 mice (the maximum relaxation at 500 µM ONOO^−^ was 81.2 ± 3.8% in intact aortae vs. 80.5 ± 4.1% in denuded aortae; *n* = 8–10; *p* = ns). Pre-incubation with 60 µM 2-APB caused a significant reduction in the relaxation to ONOO^−^ in denuded aortic rings (Fig. 1a) and this effect was also seen in endothelium-intact rings (the maximum relaxation in intact rings was 91.9 ± 10.1% vs. 65.1 ± 10.1 in the presence of 2-APB, *n* = 3 for both groups; *p* < 0.05 vs. control). Since the focus of this study was on the effects of high-fat feeding on vascular smooth muscle function, all subsequent experiments were performed in denuded aortic rings. Xestospongin C had no effect on relaxation to ONOO^−^ when added at 0.5 µM but it significantly reduced relaxation at a concentration of 5 µM (Fig. 1b). To investigate whether relaxation to ONOO^−^ involved potassium channels, several inhibitors were studied. Neither glibenclamide nor iberiotoxin had any effect, but 4-AP significantly attenuated the relaxation to ONOO^−^ (Fig. 1c).

### Effect of High-Fat Feeding on Vessel Function

**Fig. 2:**
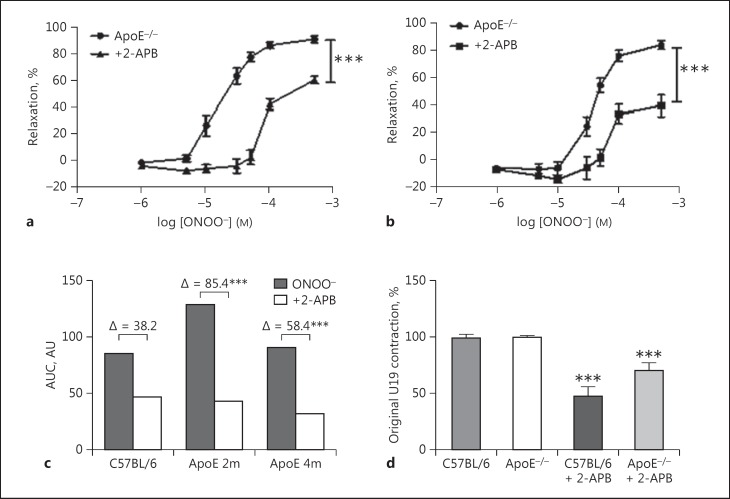
**a** In ApoE^−/−^ mice fed a high-fat diet for 2 months, 2-APB (60 µM) caused a significant attenuation of relaxation to peroxynitrite. **b** After 4 months of a high-fat diet, 2-APB still caused a significant attenuation of relaxation to peroxynitrite. **c** The degree of inhibition caused by 2-APB was compared in C57BL/6 and ApoE^−/−^ mice by measuring the area under the curves (AUC) and the difference between these areas. 2-APB had a significantly greater inhibitory effect in ApoE^−/−^ at both 2 and 4 months compared to C57BL/6 mice. **d** The addition of 2-APB reduced contraction of aortic rings to 9,11-dideoxy-9α,11α-methanoepoxy prostaglandin F_2α_ (U46619), but the effect was less marked in ApoE^−/−^ mice. 2m, 2 months of high-fat feeding; 4 m, 4 months of high-fat feeding. *** *p* < 0.001 vs. the ApoE^−/−^ control group. *n* > 5 for all groups.

In a previous study we demonstrated increased nitrotyrosine expression in the thoracic aorta of ApoE^−/−^ mice, particularly in medial and adventitial areas around atherosclerotic plaques, and this effect was more marked with time spent on a high-fat diet [6]. We hypothesized that nitration may affect smooth muscle function by modifying calcium-handling proteins within the cells and here we studied the effect of development of atherosclerotic lesions on IP3R expression and function. We found previously that the relaxation to ONOO^−^ in denuded aortic rings was largely maintained over 5 months of fat-feeding in ApoE^−/−^ mice compared to age-matched C57 controls [6] and also in ApoE^−/−^ mice fed a chow diet for 4 months (*n* = 6; data not shown). As with C57 mice, pre-incubation of ApoE^−/−^ aortic rings with 2-APB caused a significant reduction of ONOO^−^-induced relaxation. In order to compare the inhibitory effect of 2-APB in C57 and ApoE^−/−^ mice, the area under the dose-response curve in the presence and absence of 2-APB was measured using GraphPad Prism software and the difference between the two curves was calculated. This demonstrated that the degree of inhibition by 2-APB was significantly greater in mice fed a high-fat diet for 2 and 4 months compared to C57 mice. The area under the curve was 128.9 vs. 43.0 after 2 months of diet (Δ85.9; 66.6% reduction caused by 2-APB) and 90.4 vs. 32.0 after 4 months of diet (Δ58.4; 64.6% reduction caused by 2-APB) compared to 84.9 vs. 46.7 (Δ38.2; 45.0% reduction caused by 2-APB) in C57 mice (data are summarized in Fig. 2c and comparative EC_50_ and E_max_ values are given in online suppl. Table 1; see www.karger.com/doi/10.1159/000461581 for all online suppl. material). 2-APB incubation also reduced the contractile response of the aortic ring to U46619 in both C57 and ApoE^−/−^ mice with a non-significant trend towards a larger reduction in ApoE^−/−^ mice (Fig. 2d).

**Fig. 3:**
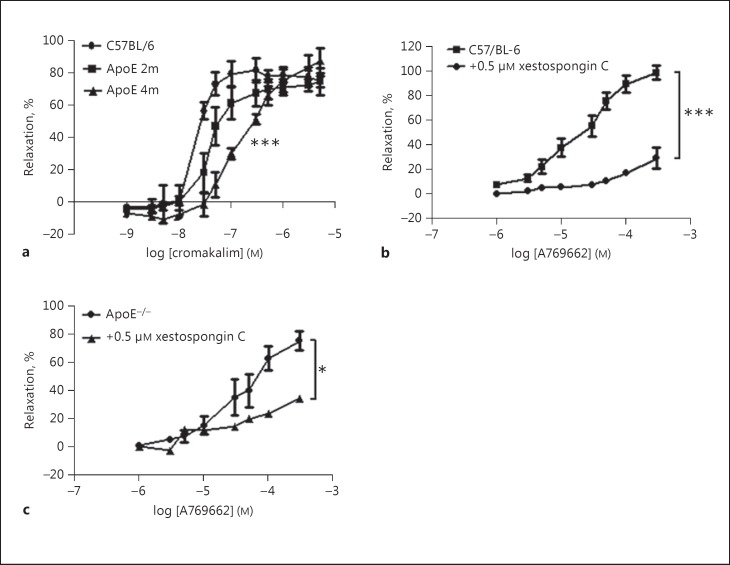
**a** The high-fat diet caused a progressive reduction in the sensitivity of aortic rings to the vasodilator cromakalim and this reached significance after 4 months of diet. *** *p* < 0.001 vs. the C57BL/6^−^ control group. **b** In C57BL/6 mice, vasodilation in response to the AMPK activating agent A769662 was attenuated by the IP3 receptor antagonist xestospongin C (5 µM). *** *p* < 0.001 vs. the C57BL/6 control group. **c** In ApoE^−/−^ mice on the diet for 4 months, relaxation to A769662 was also significantly reduced by 5 µM xestospongin C. * *p* < 0.05 vs. the ApoE^−/−^ control group. *n* > 3 for all groups. 2m, 2 months of high-fat feeding; 4 m, 4 months of high-fat feeding.

In contrast to ONOO^−^[6], the sensitivity of the aorta to relaxation by cromakalim was significantly attenuated in ApoE^−/−^ mice after 4 months of fat-feeding, with a non-significant trend towards reduced sensitivity at 2 months (Fig. 3a). A similar attenuation caused by fat-feeding on aortic relaxation to other vasodilators such as the AMPK activating agent A769662 has also been observed by us previously [19]. Relaxation to A769662 was partly dependent on IP3R activation since it was significantly attenuated by the very selective IP3R antagonist xestospongin C (5 µM) in C57 mice (Fig. 3b) and also in ApoE^−/−^ mice on the diet for 4 months (Fig. 3c).

**Fig. 4:**
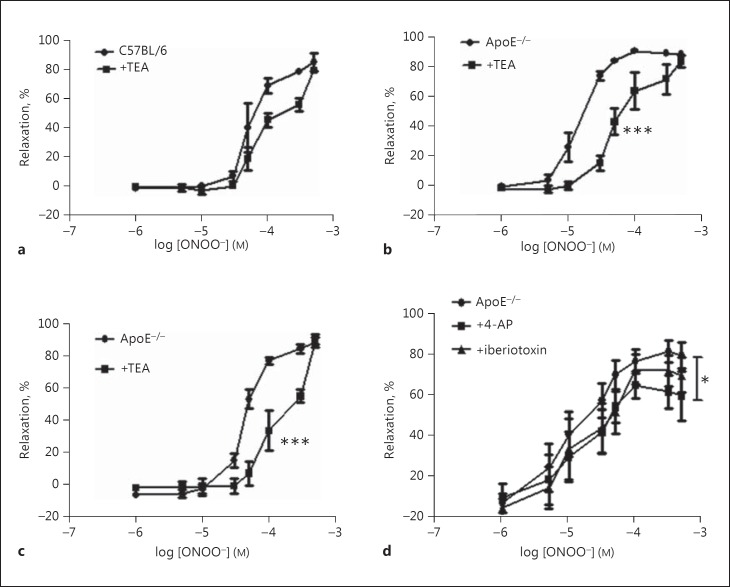
**a** Relaxation to peroxynitrite in C57BL/6 mice was not significantly different following treatment with 1 mM of the calcium-activated potassium channel blocker TEA. **b** In ApoE^−/−^ mice fed diet for 2 months, TEA caused a significant rightwards shift of the dose-response curve. **c** A significant rightward shift in response to 1 mM TEA was also observed in ApoE^−/−^ mice on a high fat diet for 4 months. **d** Iberiotoxin had no effect on relaxation in 4 month fat-fed ApoE^−/−^ mice while 4-AP significantly attenuated relaxation but to a lesser degree than that seen in C57BL/6 mice. * *p* < 0.05 and *** *p* < 0.001 vs ApoE^−/−^ control group, *n* > 3 for all groups.

In VSMC, IP3, through activation of IP3R1 can activate large-conductance calcium-activated potassium channels in the plasma membrane independently of the SR calcium release, leading to hyperpolarization and vasodilatation [4]. This may serve to limit vasoconstriction in response to IP3R activation and may underlie the attenuated vasodilation by IP3R antagonists in the current study. To test this, artery rings were treated with the non-selective calcium-activated potassium channel blocker TEA (1 mM for 30 min) and dose-response curves to ONOO^−^ repeated. TEA had no significant effect on the dose-response curve in C57 mice (Fig. 4a) but it did significantly reduce relaxation to ONOO^−^ in ApoE^−/−^ mice after 2 and 4 months on a high-fat diet (Fig. 4b, c). TEA caused a rightwards shift of the dose-response curve in ApoE^−/−^ mice with significant increases in EC_50_ and no change in E_max_ (see online suppl. Table 1). In agreement with the results in C57 mice, iberiotoxin had no effect on relaxation in 4 month fat-fed ApoE^−/−^ mice (Fig. 4d), while 4-AP significantly attenuated relaxation but to a lesser degree than that seen in C57BL/6 mice (Fig. 4d).

Together these data suggest that in mouse aorta, peroxynitrite induces relaxation in denuded vessels partly through activation of IP3R and membrane hyperpolarization via K_v_ channels.

### Histological Analysis of IP3 Expression

**Fig. 5:**
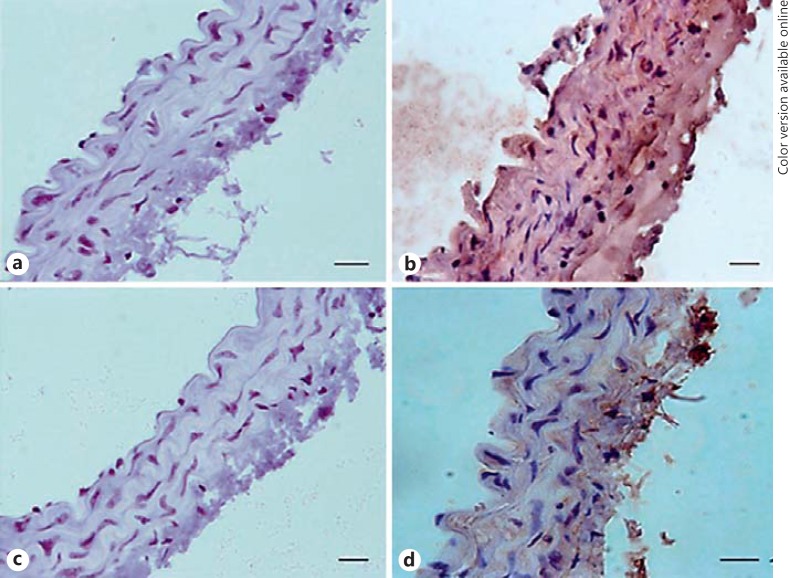
Immunostaining for IP3 receptor expression in C57BL/6 (**a**, **b**) and ApoE^−/−^ (**c**, **d**) aortic rings. Aortae were removed from ApoE^−/−^ mice on the diet for 4 months and age-matched C57 mice. No staining was visible in arteries in which the primary antibody was omitted (**a**, **c**). IP3 receptor was diffusely expressed throughout the medial layer in C57BL/6 mice (**b**), with a clear reduction in receptor expression in the high-fat-fed ApoE^−/−^ mice (**d**). Representative images are shown, with a minimum of 4 arteries studied for each group. Scale bars, 10 μm.

To study this further, we performed immunostaining for IP3R expression in paraffin-embedded samples of aorta. Only background staining was present in blank slides without antibody (Fig. 5a, b; the staining intensity was 0.013 AU in C57 sections and 0.012 in ApoE^−/−^ mice; *n* = 3). In ApoE^−/−^ mice on the diet for 4 months, the expression of IP3R1 was clearly reduced in medial SMC compared to C57 mice (Fig. 5c, d; the staining intensity averaged 0.109 AU in C57 mice vs. 0.043 AU in ApoE^−/−^ mice).

### Protein Expression/Immunoblotting

**Fig. 6:**
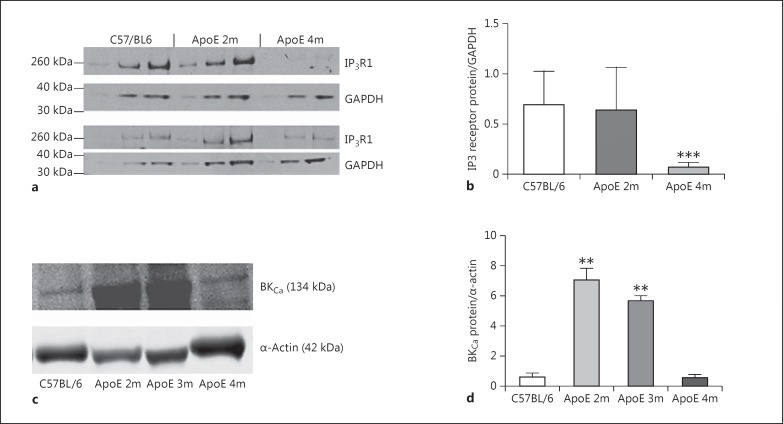
Western blotting was used to quantify the effect of a high-fat diet on IP3 and BK_Ca_ channel expression in aortic homogenates. **a**, **b** For IP3 expression, 3 different protein loads were added in adjacent lanes for each sample, with GAPDH used as the housekeeping gene. Two representative blots are shown. **b** Histogram showing a significant reduction in IP3 receptor expression after 4 months of a high-fat diet. *n* = 3. *** *p* < 0.001 vs. the C57BL/6 group. **c**, **d** For BK_Ca_ expression, α-actin was used as the housekeeping gene. **d** Histogram showing a significant increase in BK_Ca_ expression in aortic homogenates from ApoE^−/−^ on the diet for 2–3 months, with no significant change at 4 months. Representative blots are shown. *n* = 3. ** *p* < 0.01 vs. the C57BL/6 group. 2m, 2 months of high-fat feeding; 3m, 3 months of high-fat feeding; 4 m, 4 months of high-fat feeding.

To further demonstrate the reduction of IP3R expression in vascular tissue caused by fat-feeding, whole aortic homogenates were probed using antibodies to IP3R1. ApoE^−/−^ on the diet for 2 months showed no significant change in IP3R1 expression, but after 4 months on the diet there was a dramatic reduction in IP3R1 protein expression (Fig. 6a, b). Since activation of IP3R can open membrane BK_Ca_ channels to induce hyperpolarization, we measured expression of BK_Ca_ in aortic homogenates from 3 pooled samples from C57 and ApoE^−/−^ mice on the diet for 2, 3, or 4 months. Compared to C57, BK_Ca_ expression was markedly elevated in ApoE^−/−^ mice after both 2 and 3 months of fat-feeding but had declined by 4 months (Fig. 6c, d).

## Discussion

In this study we demonstrated that relaxation to peroxynitrite in endothelium-denuded mouse aortic rings is partly mediated through activation of IP3R and opening of K_v_ potassium channels. As atherosclerosis develops, the relaxation to ONOO^−^ is maintained and becomes more dependent on IP3 activation despite a reduced expression of IP3R protein and a reduced importance of the K_v_ channel. Upregulation of BK_Ca_ potassium channels may compensate for lower IP3R levels in the aorta during the early stages (2–3 months) of atherosclerosis but do not appear to be involved in vasorelaxation in C57 mice or in atherosclerotic mice after 4 months of a high-fat diet. We speculate that relaxation is maintained through increased extrusion of calcium via PMCA [6] and this maintenance of relaxation is not shared with other endothelium-independent vasodilators such as cromakalim and the AMPK activator A769662.

In all experiments, we pre-contracted aortic rings with the thromboxane A_2_ mimetic U46619. Thromboxane (and mimetics) contracts vascular smooth muscle by binding to specific G-protein-coupled receptors (TP receptors) and increasing cytosolic calcium via release from the SR. In the denuded rat pulmonary artery, contraction to U46619 was sensitive to 2-APB between 10 and 30 µM[20], similar to the findings in the present study (Fig. 2c). Interestingly, in ApoE^−/−^ mice fat-fed for 3 months, contraction to U46619 was less sensitive to 2-APB, and this is likely due to a reduced expression of IP3R in the ApoE^−/−^ aorta (Fig. 6). Contraction to U46619 was unchanged in the absence of 2-APB, suggesting that calcium release via non-IP3R-mediated mechanisms or calcium entry may sustain the contraction in the ApoE^−/−^ mouse.

In a previous study [6] also using ApoE^−/−^ mouse aortic tissue, we demonstrated compensation in calcium regulation within aortic VSMC. In ApoE^−/−^ mice, a reduction of SERCA expression was balanced by upregulation of the calcium-extruding pump PMCA, and this may explain why vasodilation to ONOO^−^ is maintained even in animals with extensive atherosclerotic lesions. Here we demonstrate that vasorelaxation to other vasodilators is not maintained. This is likely related to the mechanisms of vasodilation in response to A769772 and cromakalim. A769772 activates the enzyme AMPK in the endothelium and the VSMC and in a previous study we found that only 6 weeks of fat-feeding were sufficient to lower both total and phosphorylated AMPK expressions in the aorta compared to control mice [19]. Thus, the reduction of target enzyme levels could explain the reduced relaxation to A769772 in atherosclerotic mice. The progressively decreased sensitivity to cromakalim in ApoE^−/−^ mice on a high-fat diet for 2 and 4 months was a surprising finding. Cromakalim hyperpolarizes the SMC membrane by opening K_ATP_ channels and thereby decreasing the L-type Ca^2+^ channel opening time [21]. Previous studies have reported increases in sensitivity to cromakalim in hypertensive rats [22], while in hypercholesterolaemic rabbits dilation of the mesenteric artery in response to in vivo administration of cromakalim was preserved [23]. However, more recent studies have found that superoxide production by NADPH oxidase in the vascular wall can impair ATP-sensitive K^+^ channel function and relaxation to levcromakalim can be augmented by free radical scavengers [24]. In the ApoE^−/−^ mouse, oxidant stress in the vessel wall is progressively increased [6] and this could underlie the reduced sensitivity of the vessel to cromakalim. Since relaxation to ONOO^−^ was unaffected by the K_ATP_ antagonist glibenclamide, any impairment in the function of the K_ATP_ channel as atherosclerosis develops would not be expected to affect relaxation to ONOO^−^.

Relaxation to ONOO^−^ in the murine aorta was maintained during 5 months of fat-feeding [6] and it was attenuated by the IP3R antagonists 2-APB and xestospongin (Fig. 1, 2). In rat aortic rings, the mechanism of ONOO^−^-induced relaxation involves opening of K^+^ channels and membrane hyperpolarization [8]. We hypothesized that increases in intracellular calcium (perhaps via activation of IP3R) activate calcium-activated potassium channels on the cell membrane, leading to hyperpolarization and relaxation. In C57 aortic rings, only the K_v_ channel blocker 4-AP attenuated relaxation to peroxynitrite while the selective BK_Ca_ blocker iberiotoxin had no effect and TEA caused only a non-significant rightward shift of the dose-response curve.

In contrast, TEA significantly attenuated relaxation to ONOO^−^ in ApoE^−/−^ mice at 2 and 4 months, suggesting that opening of calcium-activated potassium channels becomes more important as atherosclerosis develops. However, TEA can also block a number of K_V_ channels and it is possible that changes in the expression of these channels were responsible for the effects seen. In support of this, iberiotoxin failed to attenuate relaxation in ApoE^−/−^ mice at 4 months and the attenuation caused by the K_V_ channel blocker 4-AP was reduced (Fig. 4d). Taken together, this indicates that although the expression of potassium channels changes during atherosclerosis, this is not responsible for maintaining the relaxation to ONOO^−^.

The lack of effect of the BK_Ca_ channel blocker iberiotoxin was somewhat surprising. Other studies have found that ONOO^−^ generates spontaneous transient outward current (STOC) via activation of BK_Ca_ channels in rat arteriolar SMC [25]. Pan et al. [25] found that an ONOO^−^ donor, i.e., SIN-1, induced a dose-dependent enhancement of STOC but 2-APB did not block the increase in STOC. They concluded that ryanodine receptor-mediated calcium release and influx from external sources activates STOC in response to ONOO^−^ and that IP3R are not involved. Conversely, in porcine coronary artery SMC, activation of IP3R with IP3 itself activated BK_Ca_ channels to enhance STOC and reduce the coronary artery tone [5].

It is possible that the regulation of potassium channels and STOC by intracellular calcium is very species specific or varies between vascular beds. Our data demonstrate that IP3R and K_v_ channels are partly responsible for the relaxation to ONOO^−^ in the mouse aorta and, as atherosclerosis develops, relaxation is maintained despite a reduced reliance on K_v_-mediated hyperpolarization. To study this further, we investigated IP3R expression in artery homogenates. Surprisingly, in view of the preserved relaxation to ONOO^−^, we found a clear and significant reduction of IP3R protein expression in ApoE^−/−^ mice after 4 months of fat-feeding (Fig. 6). Other studies have reported similar findings in VSMC exposed to oxidized LDL [26] and in atherosclerotic aorta [27]. However, in other disease states such as hypertension, there is evidence of upregulation of IP3R [28] as well as changes in the density of K^+^ currents, with a switch to predominantly BK_Ca_ currents in spontaneously hypertensive rats [29].

Since we previously demonstrated that ApoE^−/−^ mice on a high-fat diet are hypertensive [19], we speculated that an increase in BK_Ca_ channels on the aorta could compensate for the reduced IP3R expression and maintain relaxation to ONOO^−^. In pooled homogenates from 3 aortae, the expression of BK_Ca_ channel protein was markedly upregulated in ApoE^−/−^ mice on the diet for up to 3 months compared to control mice (Fig. 6). Although IP3R and BK_Ca_ channels are expressed on a wide variety of cells, the use of denuded aortae to produce the homogenates and the overwhelming propensity of VSMC in the homogenate make it likely that the changes in expression are at the level of the VSMC. If activation of IP3R is linked to STOC in the mouse aorta, upregulation of BK_Ca_ may underlie the greater inhibitory effect of 2-APB on ONOO^−^-induced relaxation in ApoE^−/−^ aortae. In support of this, isolated SMC from human atherosclerotic plaques showed evidence of a higher BK_Ca_ channel activity compared to normal medial SMC [30], and BK_Ca_ channels appear to be much less sensitive than K_v_ and K_ATP_ channels to changes caused by oxidant stress [31], making them ideal candidates to compensate for the loss of other vasodilator mechanisms in disease states [32]. However, our data with iberiotoxin indicate that BK_Ca_ is not involved in relaxation to ONOO^−^ in the C57 mouse aorta or the ApoE^−/−^ aorta after 4 months of fat-feeding. This does not rule out a role for BK_Ca_ after 2 and 3 months of fat-feeding where the expression of BK_Ca_ was upregulated; however, this seems unlikely and it appears that STOC and membrane hyperpolarization in response to ONOO^−^ is via K_V_ and that this mechanism becomes less important in atherosclerotic mice. However, it should be noted that we did not study channel activity or measure intracellular calcium and so we cannot rule out changes in intracellular calcium from sources other than IP3R in the mouse aorta.

In conclusion, our study has shown that relaxation to peroxynitrite in C57BL/6 mouse aorta is partially mediated via IP3R activation and opening of Kv potassium channels. Although atherosclerosis attenuates the response to mechanistically dissimilar vasodilators, the response to ONOO^−^ is maintained despite a progressive reduction in the expression of IP3R. Changes in the activity or expression of potassium channels during the development of atherosclerosis and increases in calcium extrusion via PMCA [11] may maintain relaxation to ONOO^−^. These compensatory changes in VSMC probably serve to limit detrimental changes in responsiveness under hyperlipidaemic conditions.

## Disclosure Statement

The authors declare that they have no conflict of interests.

## References

[1] Urena J, Fernández-Tenorio M, Porras-González C, González-Rodríguez P, Castellano A, López-Barneo J (2013). A new metabotropic role for L-type Ca^2+^ channels in vascular smooth muscle contraction. Curr Vasc Pharmacol.

[2] Leung F, Yung L, Yao X, Laher I, Huang Y (2008). Store-operated calcium entry in vascular smooth muscle. Br J Pharmacol.

[3] Narayanan D, Adebiyi A, Jaggar JH (2012). Inositol trisphosphate receptors in smooth muscle cells. Am J Physiol Heart Circ Physiol.

[4] Zhao G, Neeb ZP, Leo MD, Pachuau J, Adebiyi A, Ouyang K, Chen J, Jaggar JH (2010). Type 1 IP3 receptors activate BKCa channels via local molecular coupling in arterial smooth muscle cells. J Gen Physiol.

[5] Yang Y, Li P-Y, Cheng J, Cai F, Lei M, Tan X-Q, Li M-L, Liu Z-F, Zeng X-R (2013). IP3 decreases coronary artery tone via activating the BK Ca channel of coronary artery smooth muscle cells in pigs. Biochem Biophys Res Commun.

[6] Ewart M-A, Kennedy S, MacMillan D, Raja AL, Watt IM, Currie S (2014). Altered vascular smooth muscle function in the ApoE knockout mouse during the progression of atherosclerosis. Atherosclerosis.

[7] Gheddouchi S, Mokhtari-Soulimane N, Merzouk H, Bekhti F, Soulimane F, Guermouche B, Tani AM, Narce M (2015). Low SOD activity is associated with overproduction of peroxynitrite and nitric oxide in patients with acute coronary syndrome. Nitric Oxide.

[8] Li J, Li W, Altura BT, Altura BM (2005). Peroxynitrite-induced relaxation in isolated rat aortic rings and mechanisms of action. Toxicol Appl Pharmacol.

[9] Adachi T, Weisbrod RM, Pimentel DR, Ying J, Sharov VS, Schöneich C, Cohen RA (2004). S-glutathiolation by peroxynitrite activates SERCA during arterial relaxation by nitric oxide. Nat Med.

[10] Van Assche T, Fransen P, Guns P-J, Herman A, Bult H (2007). Altered Ca^2+^ handling of smooth muscle cells in aorta of apolipoprotein E-deficient mice before development of atherosclerotic lesions. Cell Calcium.

[11] Gleason MM, Medow M, Tulenko TN (1991). Excess membrane cholesterol alters calcium movements, cytosolic calcium levels, and membrane fluidity in arterial smooth muscle cells. Circ Res.

[12] Abou-Saleh H, Pathan AR, Daalis A, Hubrack S, Abou-Jassoum H, Al-Naeimi H, Rusch NJ, Machaca K (2013). Inositol 1,4,5-trisphosphate (IP3) receptor up-regulation in hypertension is associated with sensitization of Ca^2+^ release and vascular smooth muscle contractility. J Biol Chem.

[13] Tada T, Nawata J, Wang H, Onoue N, Zhulanqiqige D, Ito K, Sugimura K, Fukumoto Y, Shimokawa H (2008). Enhanced pulsatile pressure accelerates vascular smooth muscle migration: implications for atherogenesis of hypertension. Cardiovasc Res.

[14] Beech DJ (2012). Orai1 calcium channels in the vasculature. Pflügers Arch.

[15] Weingartner O, Husche C, Schott HF, Speer T, Bohm M, Miller CM, McCarthy F, Plat J, Lutjohann D, Laufs U (2015). Vascular effects of oxysterols and oxyphytosterols in apoE^-/-^ mice. Atherosclerosis.

[16] Cool B, Zinker B, Chiou W, Kifle L, Cao N, Perham M, Dickinson R, Adler A, Gagne G, Iyengar R (2006). Identification and characterization of a small molecule AMPK activator that treats key components of type 2 diabetes and the metabolic syndrome. Cell Metab.

[17] Miyamoto S, Izumi M, Hori M, Kobayashi M, Ozaki H, Karaki H (2000). Xestospongin C, a selective and membrane-permeable inhibitor of IP(3) receptor, attenuates the positive inotropic effect of alpha-adrenergic stimulation in guinea-pig papillary muscle. Br J Pharmacol.

[18] Huang S, Pang L (2012). Comparing statistical methods for quantifying drug sensitivity based on in vitro dose-response assays. Assay Drug Dev Technol.

[19] Greig FH, Ewart M-A, McNaughton E, Cooney J, Spickett CM, Kennedy S (2015). The hypotensive effect of acute and chronic AMP-activated protein kinase activation in normal and hyperlipidemic mice. Vascul Pharmacol.

[20] McKenzie C, MacDonald A, Shaw AM (2009). Mechanisms of U46619-induced contraction of rat pulmonary arteries in the presence and absence of the endothelium. Br J Pharmacol.

[21] Quayle JM, Bonev AD, Brayden JE, Nelson MT (1995). Pharmacology of ATP-sensitive K^+^ currents in smooth muscle cells from rabbit mesenteric artery. Am J Physiol.

[22] Kinoshita H, Kakutani T, Iranami H, Hatano Y (2001). The role of oxygen-derived free radicals in augmented relaxations to levcromakalim in the aorta from hypertensive rats. Jpn J Pharmacol.

[23] Rozsa Z, Pataricza J, Nemeth J, Papp JG (1998). Differential efficacy of vasodilators in hypercholesterolaemic rabbits. J Pharm Pharmacol.

[24] Haba M, Kinoshita H, Matsuda N, Azma T, Hama-Tomioka K, Hatakeyama N, Yamazaki M, Hatano Y (2009). Beneficial effect of propofol on arterial adenosine triphosphate-sensitive K^+^ channel function impaired by thromboxane. Anesthesiology.

[25] Pan B-X, Zhao G-l, Huang X-l, Zhao K-S (2004). Calcium mobilization is required for peroxynitrite-mediated enhancement of spontaneous transient outward currents in arteriolar smooth muscle cells. Free Radic Biol Med.

[26] Massaeli H, Austria JA, Pierce GN (2000). Overexpression of SERCA2 ATPase in vascular smooth muscle cells treated with oxidized low density lipoprotein. Mol Cell Biochem.

[27] Ewart M-A, Currie S, McCarron JG, Kennedy S (2009). Altered calcium handling between healthy and atherosclerotic vascular smooth muscle. Biophys J.

[28] Guillemette G, Bernier S (1993). Increased inositol 1,4,5-trisphosphate binding capacity in vascular smooth muscle of spontaneously hypertensive rats. Am J Hypertens.

[29] Zhang Y, Gao Y-J, Zuo J, Lee RM, Janssen LJ (2005). Alteration of arterial smooth muscle potassium channel composition and BK Ca current modulation in hypertension. Eur J Pharmacol.

[30] Wiecha J, Schläger B, Voisard R, Hannekum A, Mattfeldt T, Hombach V (1997). Ca^2+^-activated K^+^ channels in human smooth muscle cells of coronary atherosclerotic plaques and coronary media segments. Basic Res Cardiol.

[31] Terata K, Coppey L, Davidson E, Dunlap J, Gutterman D, Yorek M (1999). Acetylcholine-induced arteriolar dilation is reduced in streptozotocin-induced diabetic rats with motor nerve dysfunction. Br J Pharmacol.

[32] Liu Y, Gutterman DD (2002). The coronary circulation in diabetes: Influence of reactive oxygen species on K^+^ channel-mediated vasodilation. Vascul Pharmacol.

